# The Role of Molecular Chaperones in Virus Infection and Implications for Understanding and Treating COVID-19

**DOI:** 10.3390/jcm9113518

**Published:** 2020-10-30

**Authors:** Letizia Paladino, Alessandra Maria Vitale, Celeste Caruso Bavisotto, Everly Conway de Macario, Francesco Cappello, Alberto J.L. Macario, Antonella Marino Gammazza

**Affiliations:** 1 Department of Biomedicine, Neuroscience and Advances Diagnosis (BIND), Section of Human Anatomy, University of Palermo, 90127 Palermo, Italy; letizia.paladino91@gmail.com (L.P.); alessandra.vitale92@gmail.com (A.M.V.); celestebavisotto@gmail.com (C.C.B.); francapp@hotmail.com (F.C.); 2 Euro-Mediterranean Institute of Science and Technology (IEMEST), 90139 Palermo, Italy; econwaydemacario@som.umaryland.edu (E.C.d.M.); AJLMacario@som.umaryland.edu (A.J.L.M.); 3 Department of Microbiology and Immunology, School of Medicine, University of Maryland at Baltimore-Institute of Marine and Environmental Technology (IMET), Columbus Center, Baltimore, MD 21202, USA

**Keywords:** virus, molecular chaperones, Coronaviridae, SARS-CoV-2, COVID-19, chaperonopathies, chaperonotherapy

## Abstract

The COVID-19 pandemic made imperative the search for means to end it, which requires a knowledge of the mechanisms underpinning the multiplication and spread of its cause, the coronavirus SARS-CoV-2. Many viruses use members of the hosts’ chaperoning system to infect the target cells, replicate, and spread, and here we present illustrative examples. Unfortunately, the role of chaperones in the SARS-CoV-2 cycle is still poorly understood. In this review, we examine the interactions of various coronaviruses during their infectious cycle with chaperones in search of information useful for future research on SARS-CoV-2. We also call attention to the possible role of molecular mimicry in the development of autoimmunity and its widespread pathogenic impact in COVID-19 patients. Viral proteins share highly antigenic epitopes with human chaperones, eliciting anti-viral antibodies that crossreact with the chaperones. Both, the critical functions of chaperones in the infectious cycle of viruses and the possible role of these molecules in COVID-19 autoimmune phenomena, make clear that molecular chaperones are promising candidates for the development of antiviral strategies. These could consist of inhibiting-blocking those chaperones that are necessary for the infectious viral cycle, or those that act as autoantigens in the autoimmune reactions causing generalized destructive effects on human tissues.

## 1. Introduction

Viruses need molecules from the host cell for their survival and dissemination and molecular chaperones are among them. A timely question is about the role of molecular chaperones in the cycle of the coronavirus SARS-CoV-2, the cause of the ongoing COVID-19 pandemic.

Coronaviruses (CoVs) belong to the order Nidovirales and to the family Coronaviridae [1]. The subfamily encompasses four genera Alphacoronavirus, Betaconavirus, Gammacoronavirus, and Deltacoronavirus (α, β, γ, and δ-CoVs, respectively), and is of interest in the medical and veterinary fields. α-CoVs and β-CoVs infect mostly mammals, γ-CoVs and δ-CoVs infect birds [2,3].

CoVs are enveloped viruses with a diameter of about 100 nm with a plus-strand RNA, up to 32 kb [4]. The viral genome produces messenger RNAs (mRNAs) corresponding to the canonical structural proteins: E (envelope), M (membrane), N (nucleocapsid), and S (spike) proteins, the latter forming the distinctive corona of the viral particle [5,6].

The S protein is essential for CoVs infectivity because it contains the receptor-binding domain (RBD) and the domains involved in its fusion with the plasma-cell membrane of the host cell [7].

CoVs primarily infect the respiratory and gastrointestinal tracts of a wide range of animal species, including mammals and birds. Genetic and phylogenetic analyses have shown that CoVs have crossed species barriers frequently, even reaching humans [8,9]. CoVs infections of humans (HCoVs) seem to have originated from bat CoVs (BtCoVs). From the early 2000s to today, the world has witnessed three of these zoonotic events caused by the severe acute respiratory syndrome coronavirus (SARS-CoV), Middle East respiratory syndrome coronavirus (MERS-CoV), and severe acute respiratory syndrome coronavirus 2 (SARS-CoV-2). The latter is the most recent and has forced extraordinary measures globally, including quarantine of individuals and interruption of all kinds of human activities. SARS-CoV-2 can cause severe respiratory-tract disease and damage in various organs, such as kidneys, brain, heart, and liver with a high mortality rate [10,11,12,13,14,15]. The clinical importance of CoVs and the possibility of causing epidemics had not been duly recognized until the appearance of SARS-CoV and the MERS-CoV [16,17]. In December 2019, the new SARS-CoV-2 associated with a severe acute respiratory syndrome, quickly spread in the city of Wuhan (China), causing COVID-19 (coronavirus disease-19), affecting over 41 million people and killing over one million worldwide, at the time of this writing (latest WHO data updated October 24, 2020). This made COVID-19 a major concern for global health [18].

These events have a great negative impact on the world’s population and have forced the scientific community to quickly try to develop means for stopping infections and curing the sick [19]. Strategies for therapies may focus on the identification of the viral structures, such as the spikes with which the virus recognizes and binds to its target cells, or on the identification of the receptor in the target cell, which is recognized by the virus. Both, SARS-CoV and SARS-CoV-2 penetrate human cells using the angiotensin-converting enzyme 2 (ACE2), while dipeptidyl peptidase 4 (DPP4) represents the access receptor for MERS-CoV. Both types of receptors, which are recognized and bound by the viral spikes, are present in many tissues, such as heart, kidneys, and type 2 pulmonary alveoli [20,21,22]. It is, therefore, clear that the S protein represents a promising therapeutic target [14].

Viruses, like other infectious agents, when invading a host call into action the chaperoning (chaperone) system, which on the one side defends the infected organism and on the other it may help the attacker. The latter situation, a chaperonopathy by mistake [23], occurs when the infectious agent, such as a virus, commandeers one or more components of the chaperoning system of the infected organism and re-directs their activity to favor infection instead of counteracting its effects.

The chaperoning system is composed of molecular chaperones, co-chaperones, chaperone co-factors and chaperone interactors and receptors [23,24] that may form functional networks [25]. Molecular chaperones are proteins that have been sorted by molecular weight into various groups from the smallest (<35 kDa) to the very large (over 200 kDa) [23,26]. In some of these groups are classified molecules called Hsp (Heat Shock Protein) that belong to families of phylogenetically related proteins, such as the small Hsp with the crystallin domain, Hsp40(DnaJ), Hsp70(DnaK), and Hsp90. Although, chaperones in general are cytoprotective, when abnormal they can become pathogenic and cause disease, a chaperonopathy [27]. Chaperonopathies can be genetic or acquired with the former being the result of a genetic variant, e.g., a mutation, in the gene encoding the affected chaperone, whereas in acquired chaperonopathies the gene encoding the sick chaperone is normal but its protein product is not because it has suffered a change after transcription, for example, an aberrant post-translational modification (i.e., deacetylation, phosphorylation, or nitration). These acquired modifications may change drastically the properties of the chaperone and may be one of the mechanisms used by viruses, or cancers for instance, to make the chaperone work for their own benefit. In this situation, whereby a chaperone works for the “enemy”, so to speak, we have a chaperonopathy by mistake or collaborationism [28]. For example, it has been demonstrated that IAV (influenza A virus) induces Hsp90 acetylation leading to the nuclear import of its polymerases favouring viral replication. Therefore, Hsp90 inhibitors that block Hsp90 deacetylation could be used to treat IVA infection as well as others, such as SARS-CoV-2 [29,30].

The purpose of this review is to survey the literature and compile examples of interactions of viruses with chaperones, focusing on information that might be relevant to COVID-19, the disease caused by SARS-CoV-2, and thereby reveal points of attack for agents aimed at preventing infection, and/or blocking the chaperone-depending pathogenic mechanisms.

## 2. Molecular Chaperones and Viral Infection

Viruses are obligate intracellular parasites able to invade virtually all cells. Despite their apparent simplicity, viruses can control the host cell transcriptional/translational and metabolic machineries and the chaperoning system and use them to sustain their life cycle and carry on a productive infection.

Viruses that do not have chaperones of their own must use those of the infected host for their own sake [31]. However, many aspects of the virus-induced over expression of host’s chaperones are still incompletely understood and some are baffling, particularly because an increase of chaperoning activity may also be cytoprotective. On the one side, overexpression of some chaperones could have an antiviral effect by, for instance, stimulating an immune response against the virus, or promoting infected cells death [32,33,34], Table 1. On the other side, over expression of chaperones could favor the life cycle of the virus, including virus entry [35,36,37,38,39], and disassembly [40], nuclear import, assembly and activation of viral polymerases, as well as nuclear translocation of viral genome, and activation of replication and transcription events [41,42,43,44,45,46,47,48,49,50,51,52], and structural protein synthesis, and viral particle assembly and release [53,54,55,56]. The last steps specifically involve not only cytosolic chaperones, but also the chaperones typically expressed in the endoplasmic reticulum (ER), such as the glucose-regulated proteins (GRPs), calnexin and calreticulin [57,58], Table 1. Regarding the interplay between viruses and host cell ER, usually viral infection cause ER stress with accumulation and aggregation of misfolded proteins and activation of the unfolded protein response (UPR) [59]. UPR signaling, involving the activating transcription factor 6 (ATF6), the inositol-requiring enzyme 1 (IRE1), and the protein kinase R (PKR)-like ER kinase (PERK), induces the up-regulation of ER chaperones and other molecules required to counteract the accumulation of misfolded/unfolded proteins, including their degradation [60,61]. The UPR can be, either beneficial to viral infection because ER chaperones enhance the folding of viral proteins, or harmful because elevated expression of factors involved in protein degradation may degrade viral proteins. Therefore, to survive ER stress and UPR and to use ER chaperones for their own benefit, viruses have developed specific mechanisms [58,62,63,64]. In addition, many viruses can use the host’s chaperone system to regulate apoptosis to delay host-cell early death and promote their growth and subsequent release [65,66,67,68,69], Table 1.

Both the antiviral and the pro-infection activities of molecular chaperones present opportunities for developing anti-viral therapies, for instance by inhibiting molecular chaperones having a pro-infection activity, or by exploiting their immune activity [70,71,72,73,74].

## 3. Coronavirus and Molecular Chaperones

**Figure 1:**
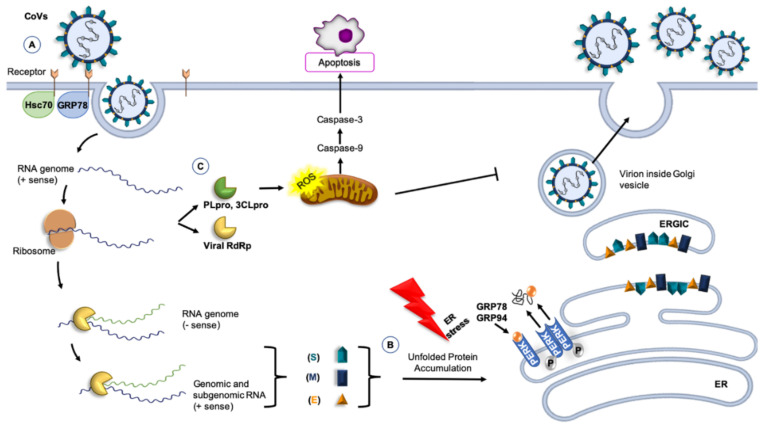
CoVs and molecular chaperones. (**A**) GRP78 or 74-kDa heat shock cognate protein 70 (Hsc70) can be part of the receptor complex recognized by the CoVs and can modulate virus entry [82,83,84]. (**B**) Molecular chaperones, such as GRP78 and GRP94, participate in the folding of CoV proteins, counteracting the effect of the stress of the host cell caused by viral infection. Accumulation of unfolded proteins induces the transcription of GRP78 and GRP94. In the endoplasmic reticulum (ER) lumen, the release of GRP78 and GRP94 from PERK are protective, inducing the unfolded protein response and controlling proteins folding. Under these conditions, ER homeostasis is restored [85]. (**C**) Non-structural proteins, such as 3C-like serine protease (3CLpro), induce apoptosis thought caspase’s pathways, causing a significant increase in reactive oxygen species (ROS) [86]. Other abbreviations: RBD, receptor-binding domain (of the S protein); PLpro, papain-like cysteine protease; RdRp, RNA-dependent RNA polymerase; PERK, PKR-like endoplasmic reticulum kinase; ERGIC, Endoplasmic reticulum-Golgi intermediate compartment.

The current understanding of the pathogenesis of HCoVs infection is still limited, but it is clear that human-to-human transmission occurs mainly within groups of people in close proximity to each other and the first critical step for virus entry into sensitive host cells involves a specific receptor [75,76]. Generally, HCoVs enters the host cell using the transmembrane spike (S) glycoprotein. This protein has two functional subunits: S1 binds the host-cell receptor and S2 is implicated in the fusion between the virus and the membrane [77]. Therefore, after attachment, the human transmembrane protease serine 2 (TMPRSS2) cleaves and activates the spike protein: S1 binds the receptor by means of its receptor binding domain (RBD) and S2 fuses the host’s membrane with the viral counterpart, an event that allows SARS-CoV-2 to enter cells by endocytosis or direct fusion of the viral envelope with the host membrane, [78,79]. In SARS-CoV and SARS-CoV-2, the receptor ACE2 is localized on the surface of smooth muscle cells; epithelia of the small intestine; respiratory tract cells, including the epithelial cells of alveoli, trachea and bronchi; and also alveolar monocytes and macrophages [76,77,80]. Furthermore, a structural analysis showed that S protein can also bind to glucose-regulated protein-78 (GRP78), an ER chaperone, on the cell surface [81], Figure 1. Instead, for MERS-CoV, the receptor named DDP4, is a surface protein widely expressed on epithelial cells in kidneys, small intestine, liver, and alveoli [21].

The S1-RBD complex mediates the fusion of the host-cell membrane and the virus with release of the viral RNA genome into the cytoplasm [82]. Once inside the cell, the RNA is then translated and transcribed, interacting with ER molecular chaperones, such as calnexin, to ensure correct protein folding, and the virus replicates [87]. Then, genomic RNA and nucleocapsid proteins combine to form new viral particles that merge with the plasma membrane to release the virus [79,88].

Since binding to receptors and membrane fusion are the initial and critical phases of the coronavirus infection cycle, one may hypothesize that a successful therapeutic strategy should target the S protein or the RBD to stop viral spread [89]. For this purpose, elucidation of the structures of the S protein and RBD would be a first and key step.

The scarce information available suggests that the level of expression of chaperones could have an impact on CoVs viral load, Figure 1. For example, the 74-kDa heat shock cognate protein 70 (Hsc70) is part of the receptor complex of the avian infectious bronchitis virus (IBV), a member of γ-CoVs [90]. A similar situation may occur for HCoVs. Also, chaperones are likely to participate in the folding of CoVs proteins and in counteracting the effects of the stress that viral infection must be causing in the host cell. The ER is a factory of proteins for secretion or membrane insertion, but an accumulation of nascent and unfolded viral proteins in the ER lumen could rapidly exceed its folding capacity and cause ER stress, which activates cell-signaling pathways to regulate gene expression at both transcriptional and translational levels [58,83,91]. This will raise the levels of chaperones in the organelle. If the damaged proteins cannot be repaired, the cell activates the mechanisms of apoptosis, but if the gene-encoding chaperones are activated and chaperones increase in ER, folding, maturation, and degradation of proteins, including viral ones, will resume, homeostasis will be restored, and viral survival will be assured [60,92].

As a result of viral replication, nascent unfolded viral polypeptides accumulate in the ER, cause ER stress by stimulating PERK and promoting mitochondrial function and metabolism [85,93]. This mechanism probably represents a viral strategy to combat the host cell’s response and to facilitate viral replication. The S protein induces the transcriptional activation of intraluminal ER chaperones, such as Hsp90β member 1 (GRP94) and binding immunoglobulin protein (Bip, also named GRP78) through PERK. The increased expression of GRP94 and GRP78 enhances the folding and processing of SARS-CoV proteins that are abundantly expressed during viral replication [93]. In addition, preclinical studies of SARS-CoV suggest that the spike glycoprotein accumulated in the ER induces the transcription of molecular chaperones, such as BiP and GRP94, leading to subsequent apoptosis in infected cells [92,94]. Therefore, the modulation of the ER by CoVs represent a new opportunity for pharmaceutical intervention. In fact, drugs are already available that modulate ER stress, inhibiting the production of infectious virions, such as in CMV infection [95]. Several studies showed that the ER stress can be suppressed through stimulation of ACE2 by the ligands angiotensin 1–7 [96,97].

In the last decade, various roles of GRP78 have been described, different from its canonical functions as an ER-residing chaperone involved in protein folding and assembly as well as in the regulation of ER stress [98]. For instance, GRP78 is also localized on the cell surface, regulating signaling and cellular homeostasis [99]. This suggests a possible role of GRP78 in virus entry when present on the surface of MERS-CoV susceptible cells [100]. Although, GRP78 is ubiquitously expressed even in cells that are not normally sensitive to the virus, it does not make non-permissive cells susceptible to MERS-CoV infection [84]. GRP78 plays an auxiliary role as an attachment factor that can modulate both MERS-CoV and SARS-CoV-2 entry in the presence of the host cell receptor DPP4 and ACE2, respectively [81]. Moreover, when DDP4 bound GRP78, the spike glycoprotein of MERS-CoV recognizes its receptor more strongly than DDP4 alone, leading to enhanced infectivity [84]. Infected Huh7 cells with MERS-CoV after siRNA knockdown of GRP78 showed a decrease of viral entry and replication, but this decrease was less marked than that caused by DPP4 knockdown [84].

In addition to the structural proteins, the coronavirus genome encodes non-structural proteins, such as papain-like cysteine protease (PLpro) and 3C-like serine protease (3CLpro) to produce RNA-dependent RNA polymerase and helicase, which are important enzymes involved in transcription and replication of CoVs [101]. PLpro and 3CLpro are attractive antiviral drug targets because of their essential function in coronavirus replication [102]. Furthermore, 3CLpro induces apoptosis through the caspase-3 and caspase-9 pathways, causing a significant increase in reactive oxygen species (ROS) [102,103]. In vivo signaling pathway assay indicated that 3CLpro increased the activation of the NF-kB dependent reporter, and proteomic analysis showed that the apoptotic factor antagonist Hsc70 was downregulated [86,103].

Since viruses take advantage of the host’s chaperoning system for the folding of their proteins and for viral assembly, targeting the pertinent member of the system is a promising strategy for developing therapies against HCoVs [104]. As discussed earlier, Hsp60 and Hsp90β promote the development and progression of viral infection, assisting virus proteins folding and genome replication. Inhibition of Hsp60 and Hsp90 expression or action (negative chaperonotherapy) could bring about the suppression of the replication of viruses, such as the influenza virus, rotavirus, and the respiratory syndrome virus [38,72,105,106]. In a recent paper, it was suggested that the pharmacological inhibition of Hsp60 could potentially ameliorate inappropriate inflammatory reaction in severe COVID-19 patients [107].

Some members of the Hsp70 family (which ones were not identified in the original report) are involved in all phases of the viral life cycle, playing an important role in regulating viruses’ proliferation, Figure 1 [108]. Therefore, the Hsp70 family inhibitors may have antiviral effects. For example, quercetin, a Bip inhibitor, has anti-porcine epidemic diarrhea virus (PEDV) capability [109]. However, since quercetin is not a specific Hsp70 inhibitor, its true value as an anti-PEDV has yet to be fully established [89].

A role of heat shock protein 40-kDa subfamily A member 1(DNAJA1) in viral infections, acting in conjunction with Hsp70, has been proposed [110], thus representing another potential target for chaperonotherapy.

**Figure 2:**
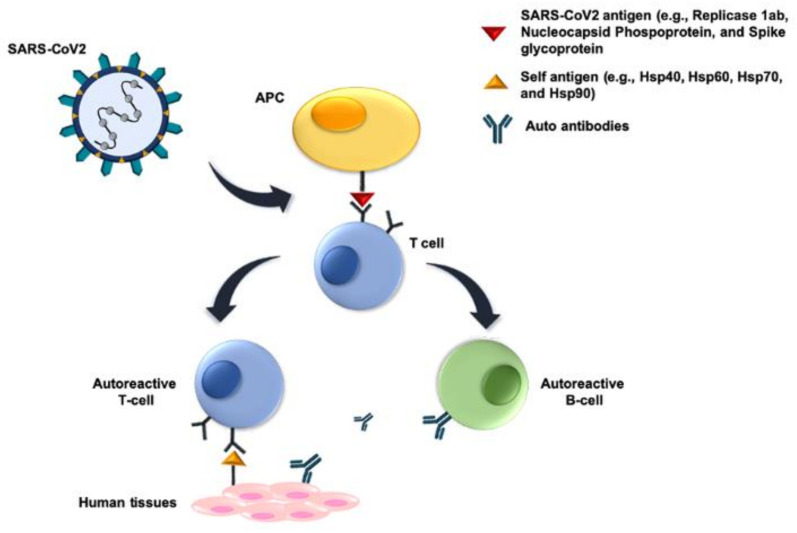
Molecular mimicry mechanisms. Molecular mimicry of host proteins by viral proteins means that there are cross-reacting antigenic epitopes shared between SARS-Cov-2 and host proteins (e.g., molecular chaperones), which induce humoral and cellular immunological reactivity against the host. The SARS-Cov-2 antigens involved probably occur in peptides of Replicase 1ab, Nucleocapsid Phosphoprotein, and Spike glycoprotein, and they induce B and/or T cells. The former cells produce antibodies reacting against the virus and also against the host crossreactive antigens. Similarly, the induced T cells react with host tissues. In both instances, autoimmunity occurs, damaging host’s tissues and organs. Abbreviation: APC, antigen-presenting cell.

A possible consequence of molecular mimicry in COVID-19 has been brought up to the fore for discussion. Viral proteins mimic human molecules, i.e., they share antigenic epitopes potentially capable of eliciting anti-viral antibodies that crossreact with the human molecules, thereby, causing autoimmune phenomena, which would be at the center of the mechanisms producing generalized vasculitis, thrombosis, and multiorgan failure [111,112,113,114,115]. Most pertinent to this review, it has been shown by bioinformatics that some human molecular chaperones share antigenic epitopes with certain SARS-CoV-2 proteins [113]. The chaperones Hsp60, Hsp90, and Hsp70 share immunogenic peptides with SARS-COV-2 proteins possibly participating in molecular mimicry phenomena after the infection (Figure 2). Cross reactive antibodies against chaperones exposed on the plasma-cell membrane of endothelial cells can lead to endothelialitis and failure of the cardiovascular system one of the pathological manifestations of COVID-19 [113,114]. In this regard, it should be mentioned that peptides shared between the virus and Hsp60 and Hsp90, are embedded in immunoreactive epitopes associated with Guillain-Barré syndrome and other autoimmune diseases such as multiple sclerosis [115]. These findings suggest the possibility of developing an immune-modulatory therapy by targeting specific molecular chaperones, to alleviate many of the symptoms and clinical consequences observed in COVID-19.

## 4. Conclusions and Perspectives

Coronaviruses, as well as other viruses, use the host’s chaperone system for the folding of their proteins and for viral assembly, translocation to the nucleus, and other vital steps Figure 2, Table 1. The host’s chaperones and their teams and networks are necessary for virus infection, survival, and spreading. It is, therefore, pertinent to classify some viral diseases within the chaperonopathies by mistake [28]. These are diseases in which normal chaperones (normal, at least to the extent that it can be determined with current technology, but we cannot exclude that they have undergone post-translational modifications that changed their properties) are etio-pathogenic factors, favoring the disease rather than protecting the organism against it. This opens the possibility of including chaperonotherapy in the therapeutic armamentarium, specifically negative chaperonotherapy, which consists of eliminating or blocking the pathogenic chaperone [81,92,116]. It is, therefore, of great interest to elucidate the role of chaperones in the SARS-CoV-2 infectious cycle, and to pinpoint chaperone-dependent steps that can be targeted by chaperone inhibitors, and the chaperones that should be eliminated or inhibited by negative chaperonotherapy. For example, chemical chaperones, such as tauroursodeoxycholic acid (TUDCA) and 4-phenyl butyric acid (PBA), have been proposed for use as therapeutic agents against the ER stress, induced by CoVs infection [92,117,118]. However, with the advances in the knowledge of the chaperoning system it becomes necessary to look at the field of chaperones and viruses with novel eyeglasses, so to speak. Chaperones do not act alone when exercising their canonical functions, namely those pertinent to protein homeostasis and quality control that are needed by virus throughout their life cycles. Therefore, studies aimed at dissecting the role of chaperones in virus biology must consider the fact that chaperones form teams (e.g., homo- or hetero-oligomers) and networks, including chaperones of various classes, co-chaperones, and chaperone co-factors to exercise their canonical functions. In addition, chaperones also have non-canonical functions, largely unrelated to protein homeostasis and quality control, but whether these non-canonical functions play any role in virus biology is still unclear. This is, precisely, another topic of great interest for exploration in the quest for a full understanding of how pathogenic viruses infect cells, and kill patients, and of how they might be defeated using methods and agents centered on components of the chaperoning system.

Also of note, is the recently posited working hypothesis incorporating molecular mimicry of human chaperones by viral proteins in the equation leading to autoimmunity and multiorgan failure in COVID-19. External agents, such as viral infections, may induce autoimmunity by the mechanism of molecular mimicry, leading to an activation of autoreactive T or B cells due to the similarities between foreign and self-peptides. For instance, some viruses, such as Epstein-Barr virus and herpesviruses, have been implicated in the development of multiple sclerosis, autoimmune encephalitis, and other autoimmune diseases [119,120]. It has been shown that 3,781 human proteins (17 of them are molecular chaperones) share peptides of at least six amino acids with SARS-CoV-2 proteins [114], opening new avenues for treatments using immunomodulatory strategies targeted to the chaperones bearing epitopes crossreactive with viral antigens.

## Figures and Tables

**Table 1:** Role of molecular chaperones in virus infections.

Mol. Chap.	Virus	Role	Reference
Hsp40	HIV-1	Participates in Nef-mediated enhancement of viral gene expression and replication	[42,46]
	IAV	Assists nuclear import of viral ribonucleoprotein	[51]
Hsp60	HBV	Participates in polymerase activation and replication initiation. Promotes virus-mediated apoptosis of infected cells	[33,41]
	HCV	Promotes infected cells apoptosis	[34]
Hsc/Hsp70	Rotaviruses	Is part of host-cell membrane receptor and promotes virus entry and infectivity	[35]
	DENV	Is part of host-cell membrane receptor and promotes virus entry and infectivity	[36]
	JEV	Is part of host-cell membrane receptor and contributes to virus entry. Enhances virus replication	[37,48]
	ZKV	Modulates virus entry, replication, and egress	[39]
	Reoviruses	Participates in outer capsid disassembly	[40]
	HCV	Regulates viral genome translation, virions assembly and release	[55]
	HIV-1	Inhibits viral gene expression. Delay Vpr induced cell cycle arrest and cell apoptosis	[46,65,66]
Hsp90	DENV	Is part of host-cell membrane receptor and promotes virus entry and infectivity	[36]
	HCV	Positively regulates virus replication	[43]
	Influenza virus	Promotes nuclear import of the genome and the RNA-dependent RNA polymerase, ensuring virus replication	[44,72]
	EBV	Mediates the assembly and the nuclear import of the DNA polymerase	[47]
	KSHV	Promotes anti-apoptotic signaling	[69]
BiP/GRP78	CMV	Regulates virions assembly	[53]
	Rotavirus	Ensures proper assembly and maturation of viral particles	[54]
GRP94	HCV	Blocks infected cells apoptosis	[68]
Calnexin	Rotavirus	Ensures proper assembly and maturation of viral particles	[54]
Calreticulin	Rotavirus	Ensures proper assembly and maturation of viral particles	[54]
CCT	Influenza virus	Promotes virus replication and transcription	[45]
	Rabies virus	Promotes virus replication and transcription	[49,50]
	DENV	Promotes virus replication and transcription	[52]
	Reovirus	Participates in viral proteins folding	[56]

Abbreviations: Mol. Chap, molecular chaperone; CMV, cytomegalovirus; DENV, dengue virus; EBV, Epstein-Barr virus; HBV, hepatitis B virus; HCV, hepatitis C virus; HIV-1, human immunodeficiency virus type 1; IAV, influenza A virus; JEV, Japanese encephalitis virus; KSHV, Kaposi’s sarcoma–associated herpesvirus; ZKV, Zika virus.
